# Cross-Modal Stochastic Resonance as a Universal Principle to Enhance Sensory Processing

**DOI:** 10.3389/fnins.2018.00578

**Published:** 2018-08-21

**Authors:** Patrick Krauss, Konstantin Tziridis, Achim Schilling, Holger Schulze

**Affiliations:** Department of Otorhinolaryngology, Head and Neck Surgery, Experimental Otolaryngology, Friedrich-Alexander University Erlangen-Nürnberg, Erlangen, Germany

**Keywords:** stochastic resonance, cross-modal perception, neural processing, dorsal cochlear nucleus, tinnitus

Cross-modal interactions are common in sensory processing, and phenomena reach from changed perception within one modality due to input from another like in the well-known McGurk effect (McGurk and MacDonald, 1976) to misperceptions like synesthesia (Marks, 1975). A recent study demonstrated that electro-tactile stimulation applied to the index finger significantly improves speech perception thresholds (Huang et al., 2017). Here we argue that such cross-modal enhancement can be explained in terms of stochastic resonance (Benzi et al., 1981), a phenomenon that is ubiquitous in nature (Wiesenfeld and Moss, 1995), and especially within the context of neuroscience, receives increasing attention (Douglass et al., 1993; Moss et al., 2004; Faisal et al., 2008; Mino, 2014).

Stochastic resonance refers to a processing principle where signals otherwise sub-threshold for a given sensor can, at least partially, be detected anyway by adding noise of a suitable intensity to the sensor input (Benzi et al., 1981; Collins et al., 1996; Levin and Miller, 1996; Gammaitoni et al., 1998).

In self-adaptive signal detection systems based on stochastic resonance, the optimum noise level is continuously adjusted via a feed-back loop, so that the system response in terms of information throughput remains optimal, even if the properties of the input signal change. For this processing principle the term adaptive stochastic resonance has been coined (Mitaim and Kosko, 1998, 2004; Wenning and Obermayer, 2003). Most objective functions to quantify such information transmission, e.g., mutual information, require knowledge of the signal to be detected (Levin and Miller, 1996; Mitaim and Kosko, 2004; Moss et al., 2004). In a previous study we demonstrated that the autocorrelation of the sensor output, a quantity always accessible and easy to analyze by neuronal nets, can be used to quantify and hence maximize information transmission even for unknown and variable input signals (Krauss et al., 2017). In a further study we demonstrated by implementing a phenomenological computational model that adaptive stochastic resonance based on output autocorrelations might be a major processing principle of the auditory system (Krauss et al., 2016) that serves to partially compensate for acute or chronic hearing loss, e.g., due to cochlear damage (Krauss et al., 2016; Gollnast et al., 2017). In that view, the noise necessary for stochastic resonance to work corresponds to increased spontaneous neuronal firing rates in early processing stages of the auditory brainstem and cortex, a phenomenon that has been observed in animal models and human subjects with central tinnitus (Wang et al., 1997; Ahlf et al., 2012; Tziridis et al., 2015; Wu et al., 2015). In fact we have proposed earlier that the noise necessary for stochastic resonance is the neurophysiological correlate of tinnitus-related enhanced neuronal activity, and that in this view tinnitus is a side effect of an adaptive mechanism within the auditory system whose main purpose is to compensate for hearing loss by constantly optimizing information transmission (Krauss et al., 2016).

Within the auditory system, the dorsal cochlear nucleus (DCN) is the earliest processing stage in the auditory pathway in which acoustic trauma leads to tinnitus-related changes and increased spontaneous firing rates (Kaltenbach et al., 1998, 2004; Kaltenbach and Afman, 2000; Brozoski et al., 2002; Zacharek et al., 2002; Wu et al., 2015). The amount of this increase in spontaneous activity in the DCN has been shown to be correlated with the strength of the behavioral signs of tinnitus (Kaltenbach et al., 2004). Furthermore, this hyperactivity is only found in regions innervated by the damaged parts of the cochlear receptor epithelium (Kaltenbach et al., 2002). Gao and colleagues recently described changes in DCN fusiform cell spontaneous activity after noise exposure that supports our proposed stochastic resonance mechanism. In particular, the time course of spontaneous rate changes shows an almost complete loss of spontaneous activity immediately after loud sound exposure (as no stochastic resonance is needed due to stimulation that is well above threshold), followed by an overcompensation of spontaneous rates to levels well above pre-exposition rates where stochastic resonance is now needed to compensate for acute hearing loss (Gao et al., 2016).

It is well-known that the DCN receives not only input from the cochlea, but also from the somatosensory system (Ryugo et al., 2003; Shore and Zhou, 2006; Dehmel et al., 2012; Zeng et al., 2012). Therefore, in a previous paper we proposed the possibility that the neuronal noise which is crucial for stochastic resonance to work may be injected into the auditory system via somatosensory projections (Krauss et al., 2016). This idea is supported by a number of papers.

For example, it is well-known that jaw movements lead to a modulation of tinnitus sensation in patients (Pinchoff et al., 1998). This may easily be explained within our model, as jaw movements alter somatosensory input to the DCN. Since this somatosensory input would correspond to the noise which is crucial for stochastic resonance, auditory input to the DCN is modulated through this mechanism and the altered noise level would be perceived as modulated tinnitus (Krauss et al., 2016). Tang et al. demonstrated that somatosensory (noise) input and hence tinnitus sensation may also be modified by serotonergic regulation of excitability of principal cells of the DCN (Tang and Trussell, 2015, 2017).

In addition, the finding that DCN responses to somatosensory stimulation are enhanced after noise-induced hearing loss (Shore et al., 2008) supports our idea that stochastic resonance plays a key role in auditory processing and actually takes place in the DCN. After hearing-loss, the auditory input to the DCN is decreased. By that, information transmission is reduced leading to an increase of internally generated neuronal noise which is crucial for adaptive stochastic resonance to work and partially restore hearing thresholds.

Finally, the finding that electro-tactile stimulation applied to the index finger significantly improves speech perception thresholds (Huang et al., 2017) further supports our interpretation that somatosensory input to the DCN corresponds to the noise input for stochastic resonance. Again, electro-tactile stimulation increases somatosensory input which is equivalent to increased neuronal noise, which in turn improves detection thresholds for auditory stimuli via stochastic resonance.

In a previous paper we already discussed the possibility of superseding the internally generated neuronal noise by adding external acoustic noise, thereby suppressing the tinnitus percept (Krauss et al., 2016). Remarkably, a recent publication demonstrated that exposure to moderate levels of white noise after acoustic trauma prevented most individuals from developing tinnitus in a mouse model (Sturm et al., 2017). Along the same line, Marks and coworkers found that auditory-somatosensory bimodal stimulation reduces tinnitus in guinea pigs and humans (Marks et al., 2018). Within the framework of our modal one can argue that simultaneous auditory and somatosensory stimulation corresponds to a combination of the two aforementioned studies (Huang et al., 2017; Sturm et al., 2017).

Based on these observations we here speculate that stochastic resonance in one sensory modality driven by input from another modality may be a general principle, namely multisensory integration causing stochastic resonance like cross-modal enhancement. For example, it is known that the concept of stochastic resonance via internal noise applies also to visual perception (Aihara et al., 2008). Furthermore, analogous to the above discussed somatosensory enhancement of auditory perception, visual perception at threshold can be enhanced by auditory stimulation (Caclin et al., 2011) and that even visual below threshold stimuli may be perceived through spatially converging audiovisual inputs (Bolognini et al., 2005). Such audiovisual cross-modal improvement of detection thresholds has been demonstrated in a broad range of different species, e.g., the ferret (Hollensteiner et al., 2015) or the chicken (Verhaal and Luksch, 2016).

In addition to the here proposed neuronal mechanism of somatosensory-driven stochastic resonance in the auditory system explaining the better hearing in the CI patients during finger tapping (Huang et al., 2017), alternative explanations are discussed: First, an attentional effect modulating the sensory threshold has been proposed (for Review see Sarter et al., 2005). Second, due to the fact that patients are dealing with a multimodal integration task, the interaction of both stimuli can mathematically be explained by the “optimal observer” model of sensory integration based on maximum-likelihood estimation theory as already shown in visual and auditory (e.g., Battaglia et al., 2003) and visual and somatosensory interaction (Hollensteiner et al., 2015). Nevertheless, both alternative interpretations would most likely interact on the level of cortical representations of the sensory modalities. Hence, to proof or falsify our hypothesis proposed here that auditory-somatosensory interaction takes place at the level of the dorsal cochlear nucleus, neuronal recordings in the dorsal cochlear nucleus should be performed in search of a somatosensory driven modulation of auditory neuron spontaneous activity in combination with lowered neuronal and/or behavioral thresholds, Nevertheless, finding such interaction within the DCN would not disproof that cross-modal interactions between other modalities and/or in other contexts might take place at the level of sensory cortex.

Even though multisensory integration leading to improved perception has been reported earlier (Stein and Stanford, 2008), we here provide a new mechanistic explanation of how this enhancement might work. We hypothesize that this mechanism represents a universal principle of neural computation.

## Author contributions

PK and HS wrote the manuscript. PK, HS, KT, and AS discussed possible interpretations of the cited studies and developed the hypothesis presented in this paper.

### Conflict of interest statement

The authors declare that the research was conducted in the absence of any commercial or financial relationships that could be construed as a potential conflict of interest. The reviewer SV and the handling editor declared their shared affiliation.
